# The history of families at-risk for hereditary breast and ovarian cancer: what are the impacts of genetic counseling and testing?

**DOI:** 10.3389/fpsyg.2024.1306388

**Published:** 2024-03-04

**Authors:** Natalia Campacci, Rebeca Silveira Grasel, Henrique de Campos Reis Galvão, Lucas França Garcia, Paula Carvalho Ribeiro, Kercy Fram de Jesus de Sena Pereira, José Roberto Goldim, Patricia Ashton-Prolla, Edenir Inêz Palmero

**Affiliations:** ^1^Molecular Oncology Research Center, Barretos Cancer Hospital, Barretos, Brazil; ^2^Genomic Medicine Service, Hospital A Beneficência Portuguesa de São Paulo, São Paulo, Brazil; ^3^Department of Genetics, Brazilian National Cancer Institute, Rio de Janeiro, Brazil; ^4^Oncogenetics Department, Barretos Cancer Hospital, Barretos, Brazil; ^5^Centro Universitário Cesumar, Maringá, Brazil; ^6^Hospital de Clínicas de Porto Alegre, Porto Alegre, Brazil

**Keywords:** genetics, hereditary cancer, genetic counseling, breast and ovarian cancer predisposition syndrome, family dynamics

## Abstract

**Introduction:**

Cancer Genetic Counseling (CGC) and genetic testing (GT) assume a paramount role for hereditary cancer predisposition syndrome families. We assessed the effects of CGC and GT on women affected by cancer who are at risk for hereditary breast and ovarian cancer predisposition syndrome (HBOC).

**Methods:**

This study encompasses four time points: before the CGC session, after the CGC session when blood is drawn for GT, after disclosure of GT results, and six months following disclosure of GT results. The impacts of CGC and GT were assessed using psychosocial questionnaires. Additionally, a pedigree, genogram, and ecomap were constructed through a semistructured interview.

**Results:**

A total of sixty women were included in the study. Most participants considered their perception of cancer risk to be equivalent to that of the general population, even among those with pathogenic variants. An increased perception of breast and ovarian cancer risks was associated with a heightened inclination toward religious engagement as a coping mechanism. Patients carrying variants of uncertain significance expressed greater concerns about developing another cancer compared to those who had *BRCA1* and *BRCA2* wild type or pathogenic variants. Qualitative analysis of the genograms and ecomaps demonstrated that the CGC/GT processes facilitate communication within families. The genogram analyses revealed the impact of CGC and GT processes on families at risk for hereditary cancer. Changes in some family relationships were observed, and an improvement in communication was noted following the GT process.

**Discussion:**

These findings can assist healthcare professionals considering a personalized approaches in clinical practice.

## Introduction

Ever since the discovery that women harboring germline pathogenic variants in the *BRCA1* and/or *BRCA2* genes face elevated risks of developing breast and ovarian cancer over their lifetime, coupled with the critical need to appraise new treatment modalities for those under cancer diagnoses, the demand for *BRCA* testing has seen a surge (Forbes et al., 2019), leading to the identification of many families with Hereditary Breast and Ovarian Cancer Syndrome (HBOC), an inherited disorder in which the risk for breast, ovarian, pancreatic and prostate cancer is higher (National Cancer Institute Dictionary of Cancer Terms, 2023). Hence, the Cancer Genetic Counseling (CGC) process assumes paramount importance, serving as a pivotal step in aiding these women and their family members in comprehending and adapting to the medical, psychological, and familial implications associated with their genetic disposition. Furthermore, it stands as an indispensable educational and supportive component, instrumental in facilitating their decision-making processes (Hampel et al., 2015; Bashford et al., 2019).

Given the significance of CGC, the entire spectrum of processes may potentially give rise to discomforting emotions, encompassing anxiety and depression (Mella et al., 2017), distress stemming from the fear of the unknown (Dorval et al., 2008), and/or other psychological challenges (Eijzenga et al., 2015; Cicero et al., 2017). Irrespective of the outcome, a genetic test (GT) carries profound implications, particularly for women harboring pathogenic variants in *BRCA1* and *BRCA2*, often culminating in heightened psychological stress and diminished coping capacity in response to the GT results (O’Neill et al., 2015). Furthermore, a negative result, when experienced by an individual in a positive-testing family, may evoke feelings of guilt and exclusion (Douglas et al., 2009). Variants of uncertain significance (VUS) also introduce complexities into CGC, as patients grappling with such outcomes may struggle to fathom the ramifications of VUS on their personal lives and the lives of their kin (Hallowell et al., 2002).

Hamilton et al. described in a qualitative and longitudinal study that there was significant changes in daily life and health behavior decisions made 3 to 4 years after undergoing GT for HBOC but without cancer diagnosis, showing that there are positives and negatives consequences in long term (Hamilton et al., 2009). Coping strategies with respect to GT exhibit heterogeneity, potentially giving rise to a plethora of shifts within family dynamics, including difficulties in family communication and relationships (Douglas et al., 2009). It is well-established that family communication assumes a pivotal role in aiding family members who themselves have not undergone CGC in navigating their choices to mitigate their cancer risk (Riley et al., 2012).

Individuals who have already received a cancer diagnosis are navigating a spectrum of emotionally vulnerable states. Introducing the complexities associated with undergoing CGC and GT processes may further complicate the management of their own well-being and that of their family members. The way individuals experience CGC and the implications of a GT result are highly individualized and may be influenced by personal experiences and the sociocultural context each individual has experienced (Gilbar et al., 2016).

Gaining insights into how individuals deal with CGC and GT, as well as their perception of these processes regarding risk, concerns, and health and screening beliefs in long term, can serve to equip counselors with enhanced tools for delivering more effective CGC and mitigating potential adverse effects inherent in this process. The literature has highlighted several important tools designed to enhance our understanding of the psychological and psychosocial impacts of the CGC process. However, it is crucial to utilize tools that have undergone translation, cultural adaptation, and validation processes to ensure greater accuracy of the obtained data (Sousa and Rojjanasrirat, 2011).

In this sense, the present study seeks to assess the impact of CGC and GT, on cancer-affected women who are at risk for hereditary breast and ovarian cancer, along with their families, spanning from the inception of the CGC process to a six-month juncture after the disclosure of the GT outcomes by using psychosocial questionnaires and the construction of pedigree, genogram and ecomap.

## Materials and methods

### Participants and study scenario

The study cohort was referred from the oncological treatment department to the oncogenetics department (OD) of a prominent cancer reference hospital in Brazil. The operational framework of the OD entails an initial appointment for CGC, with two parts: the first part conducted by a nurse, involving the collection of sociodemographic data, patients’ cancer history, and pertinent familial information to construct pedigrees; the second part comprises a consultation with a medical geneticist, wherein the GT and its implications are deliberated upon. Exhaustive details regarding all OD processes and protocols have been documented in prior publications (Palmero et al., 2016; Campacci et al., 2020). A total of 60 women were included and responded to all questionnaires. They had previously received a breast and/or ovarian cancer diagnosis, were above the age of 18, and exhibited a family history (FH) with criteria for breast and ovarian cancer predisposition syndrome (HBOC), aligning with the GT criteria stipulated by the NCCN guidelines (National Comprehensive Cancer Network, 2019). The sample was entirely composed by women due to the reality of the OD where the study was conducted and to the higher prevalence of breast cancer among women. Furthermore, for qualitative analysis, the analyses would be standardized, since female health issues may differ from male issues.

The determination of the sample size was performed on the historical attendance of patients at the OD during preceding years, encompassing those with both personal and FH traits suggestive of HBOC. *A posteriori* power analysis was performed using Gpower 3.0.10 to determine whether the number of included patients would be representative of the true OD attendance. Based on the calculations, considering an error rate of 0.05, a power of 0.8, and an effect size of 0.18, an approximate sample size of 60 was deemed appropriate.

### Psychological assessment

The instruments were meticulously chosen to assess various dimensions of the study, encompassing patients’ perception of cancer risk (Cancer Risk Perception Scale – CANS) (MacDonald et al., 2002), apprehensions regarding the potential development of another cancer (Cancer Worry Scale – CWS) (Lerman et al., 1994), constructs from the Health Belief Model (CHBM) (Champion, 1984), strategies employed for coping (Everyday Memory Problems – EMEP) (Seidl et al., 2001), levels of anxiety and depression (Hospital Anxiety and Depression Scale – HADS) (Zigmond and Snaith, 1983), and the dynamics of family history (utilizing genograms and ecomaps) (McGoldrick and Gerson, 1999). It is imperative to note that the rationale for choosing the instruments was based on the previously undergone validation procedures for their Brazilian-Portuguese language iterations (Zigmond and Snaith, 1983; Botega et al., 1995; McGoldrick and Gerson, 1999; Seidl et al., 2001; Santos, 2008; Silva et al., 2013). For an in-depth understanding of the measurement methodologies applied to these psychological instruments, a comprehensive exposition is provided in the Supplementary Table S1.

All psychosocial instruments were printed on sheets of paper and administered through face-to-face interviews at the OD clinic. At T0 and T3, the interviews, lasted an average of 25 min, while at T1 and T2 they lasted an average of 15 min. All collected data were recorded in the database using IBM SPSS Statistics 22 for analysis. As for the semi-structured interviews (Supplementary Table S2), they were conducted by the oncogenetics nurse (NC), who had received communication training in qualitative studies and genetic counseling in a clinical setting at OD. Each interview had an approximate duration of 30 min.

### Study overview

During the initial appointment, prior to the CGC process, women were invited by a genetic nurse (NC) to participate in the study. All 60 participants consented to participate and provided questionnaire and narrative data through a semi-structured interview. The sequential phases of the study were as follows:

T0: Before the CGC session, conducted by the oncogenetics nurse. During this phase, a genogram and an ecomap were constructed, supplemented by a semi-structured interview using an interview form validated by Wright and Leahey (1999) (refer to Supplementary Table S2). Additionally, an array of assessment tools, including the CWS, CANS, CHBM, EMEP, and HADS, were employed.
T1: After the CGC session when blood was drawn for genetic testing (GT), conducted on the same day as T0 but a few hours later. During this phase, the CWS, HADS, and CANS tools were applied.
T2: After the disclosure of GT results, with the application of the CWS, HADS, and CANS tools. This milestone was reached 3 months subsequent to T1.
T3: Six months following the disclosure of GT results. At this stage, the pedigree, genogram, and ecomap were revisited, along with a follow-up semi-structured interview (refer to Supplementary Table S2). Additionally, a reassessment using the CWS, CANS, CHBM, EMEP, and HADS tools was carried out.

### Data analysis

Data analysis adopted a mixed-methods approach to comprehensively examine the changes transpiring during the course of the CGC and GT processes, encompassing both quantitative and qualitative analyses.

Quantitative Analysis: Quantitative data were summarized using measures such as mean, standard deviation, minimum, and maximum values for numerical variables, while categorical variables were presented as frequencies and percentages. For categorical variables, comparisons were undertaken employing the Chi-squared test or Fisher’s exact test. For numerical variables, normality-based considerations led to the utilization of either Student’s *t*-test and ANOVA or the Mann–Whitney test and Kruskal-Wallis test. To explore patterns of change within responses to the CANS, CWS, and HADS questionnaires, the Marginal Homogeneity Test was employed. To assess the internal consistency of the CWS, CHBMS and EMEP questionnaires, Cronbach’s α was computed, with values exceeding 0.7 indicative of satisfactory reliability (Cronbach and Meehl, 1955). The information about Cronbach’s α from the moments of the study are at Supplementary Tables S3–S5. Statistical analyses were conducted using IBM SPSS Statistics 22.

Qualitative Analysis: The qualitative component encompassing genograms, ecomaps and semistructured interviews underwent an intricate qualitative analysis. Employing content analysis within Laurence Bardin’s thematic or categorical analysis framework (Bardin, 2016), a comparison between outcomes from T0 and T3 was established. The software NVivo V.11Pro was employed to support the qualitative analysis. The analyses were dual-faceted, orchestrated by two professionals: a nurse who conducted all of the interviews (NC) and a separate researcher (a social scientist) who maintained an uninvolved stance in the data collection (LG), thus enriching the observational insights. The qualitative data were subjected to interpretation guided by the “life course perspective” theory, which scrutinizes an individual’s life within their contemporary social context. Given the influence of family history and age on health-related decisions and event perceptions, this theoretical framework was harnessed to dissect the data. Age, familial cancer history, and GT outcomes were scrutinized within this study (Hutchison, 2005).

## Results

### Quantitative analysis

**Figure 1:**
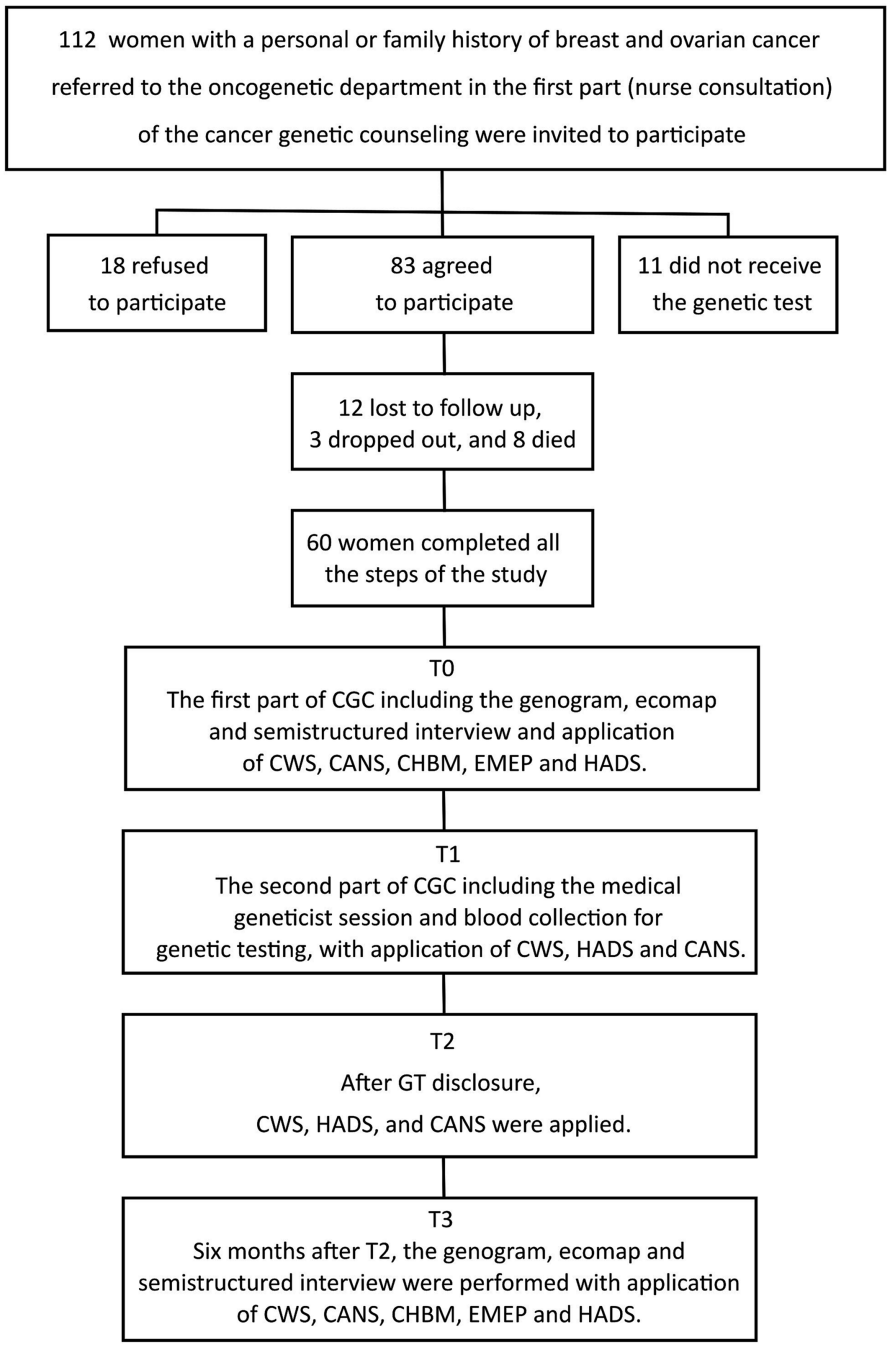
Study procedures and patient enrollment.

Figure 1 illustrates a comprehensive flowchart delineating the study’s design and the trajectory of patient recruitment. In brief, a total of 112 women were extended invitations to participate in the study; nonetheless, the study was ultimately carried forward with a cohort of 60 women participants who expressed consent and diligently navigated through each stipulated time point within the study protocol. Within the final assemblage, 41 patients exhibited no germline pathogenic variants (WT – negative GT) in *BRCA1*, *BRCA2*, or *TP53*, while 16 participants yielded a positive GT outcome (MT), encompassing 8 individuals with germline pathogenic variants in *BRCA1*, 2 with variants in *BRCA2*, and 6 individuals with variants in *TP53*. Additionally, 3 patients were identified as harboring variants of uncertain significance (VUS), with 1 VUS in *BRCA1* and 2 VUS in *BRCA2*. The sociodemographic data, presented in Table 1, indicate that the participants had a mean age of 42.5 years (SD = 10). Most participants had a diagnosis of breast cancer (83.3%), a family history of cancer (93.3%), and reported having children (85%).

**Table 1:** Sociodemographic information of the sample.

	*N* (%)	Mean (±SD)	Minimum	Maximum
Sex				
-Female	60 (100)	–	–	–
Education				
-Primary school	11 (18.3)	–	–	–
-Secondary school	25 (41.7)	–	–	–
-Graduate	24 (40)	–	–	–
Marital status				
-Single	12 (20)	–	–	–
-Married	41 (68.3)	–	–	–
-Divorced	2 (3.3)	–	–	–
-Widow	5 (8.3)	–	–	–
Have a job				
-No	13 (21.7)	–	–	–
-Yes	47 (78.3)	–	–	–
Cancer				
-Breast cancer	50 (83.3)	–	–	–
-Bilateral breast cancer	4 (6.7)	–	–	–
-Breast and gastric cancer	1 (1.7)	–	–	–
-Breast and colorectal cancer	2 (3.3)	–	–	–
-Breast and ovarian cancer	1 (1.7)	–	–	–
-Ovarian cancer	2 (3.3)	–	–	–
Surgery				
-Mammary surgery with curative intention	48 (80)	–	–	–
-Oophorectomy with curative intention	3 (5)			
-Oophorectomy for other gynecological problems	9 (15)	–	–	–
Family history of cancer				
-Yes	56 (93.3)	2.9 (±1.8)	0	6
-No	4 (6.7)			
Have children				
-Yes	51 (85)	1.67 (±1.1)	0	5
-No	9 (15)			

### Cancer risk perception

**Figure 2:**
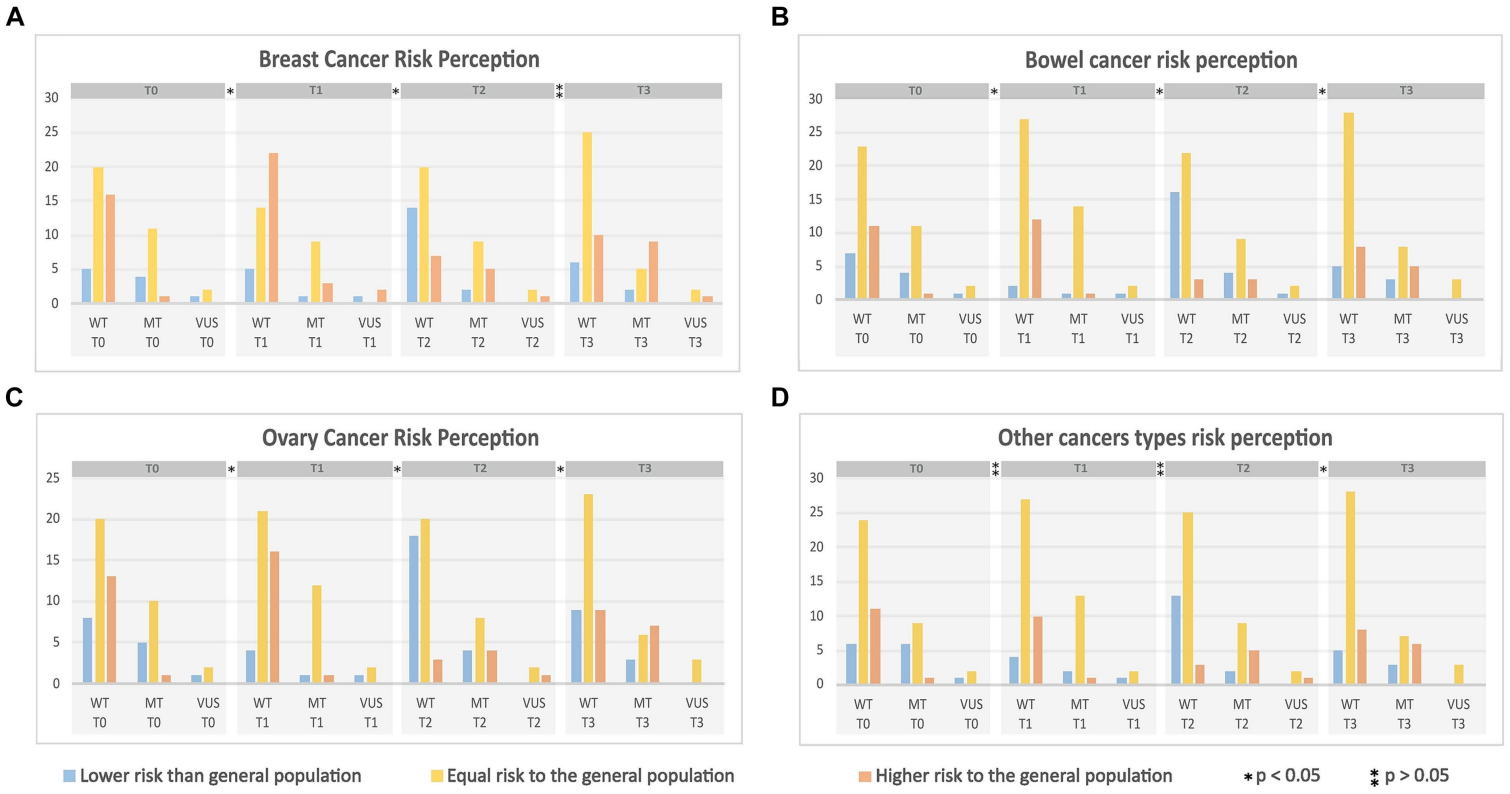
Results of the CAN application—For each type of cancer, the risk perception is demonstrating through the different assessment time point separated by genetic test result status [MT- (mutation) is the presence of a pathogenic variant; WT- (wild type gene) is the absence of a pathogenic variant; VUS- (variant of uncertain significance) is the presence of a variant of unknown significance]. **(A)** The breast cancer risk perception through the time points of the study and for those with * (*p* < 0.05) shows that process of CGC and GT can elevate or diminishing breast cancer risk perception. **(B)** The bowel cancer risk perception through the time points of the study and for those with * (*p* < 0.05) shows that process of CGC and GT can elevate or diminishing breast cancer risk perception. **(C)** The ovary cancer risk perception through the time points of the study and for those with * (*p* < 0.05) shows that process of CGC and GT can elevate or diminishing breast cancer risk perception. **(D)** The other cancer types risk perception through the time points of the study and for those with * (*p* < 0.05) shows that process of CGC and GT can elevate or diminishing breast cancer risk perception.

Throughout all phases of the study, the majority of women considered their cancer risk equal to that of the general population, as evidenced by the CAN questionnaire (refer to Figure 2), even among those harboring pathogenic variants (MT). Notably, the responses of some women underwent modifications during the CGC and GT sequences. This fact was evaluated by the marginal homogeneity test, which showed the statistical significance (*p* < 0.05) in the evolving response patterns, both in terms of elevating and diminishing cancer risk perception over the successive phases.

Several variables, including surgical interventions (mastectomy and/or salpingo-oophorectomy), educational attainment, familial cancer history, and GT outcomes, were meticulously evaluated to ascertain their potential influence on cancer risk perception. The impact of the GT outcome surfaced as significant (*p* = 0.048) during T2 (following the reception of GT results), particularly concerning the “perception of risk for other cancers.” This perception of heightened risk for alternative cancer types emerged predominantly among women with presence of pathogenic variants results. Furthermore, the undertaking of salpingo-oophorectomy reverberated with an effect on cancer risk perception across all time points (*p* < 0.05).

### Worry about developing a new cancer

**Figure 3:**
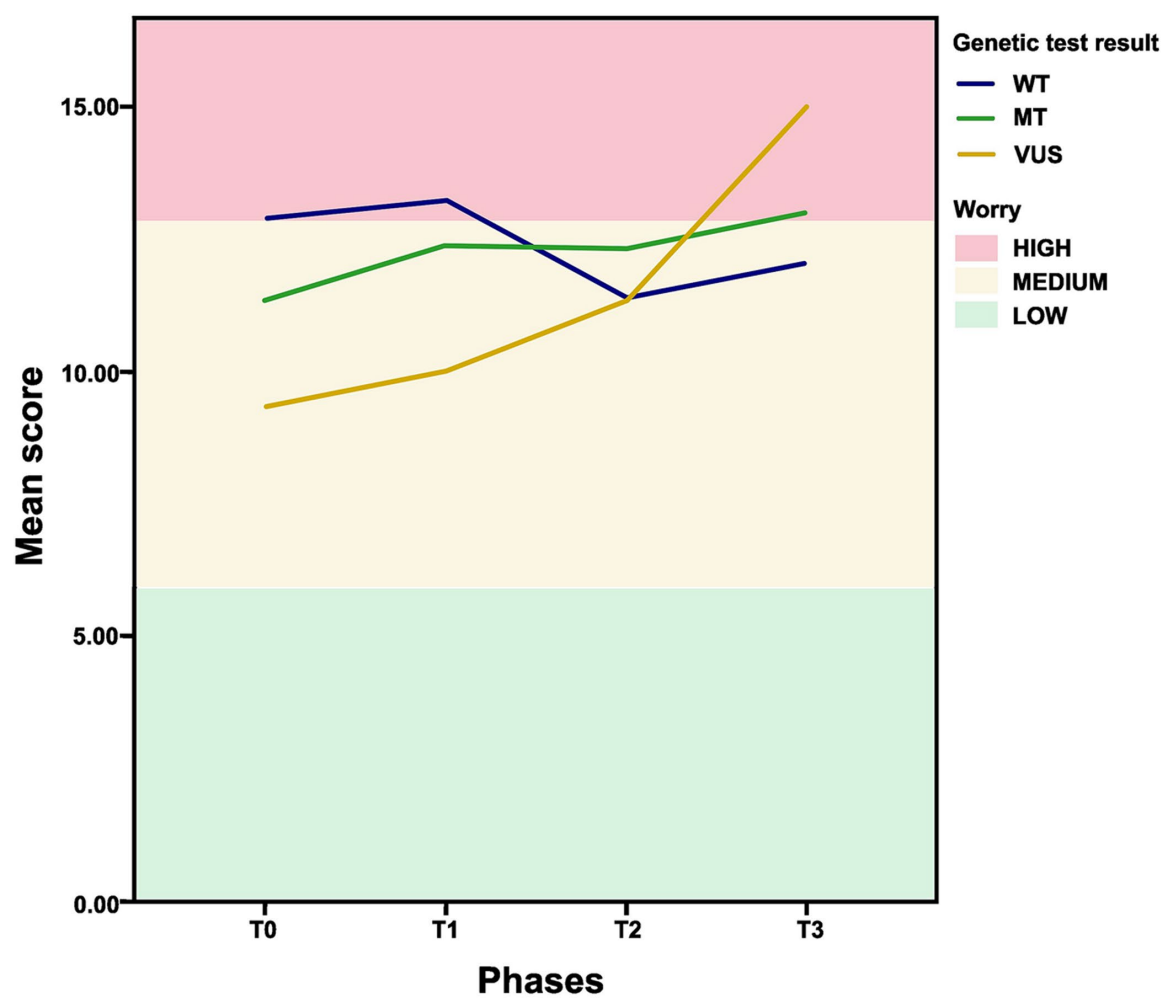
Cancer worry to develop cancer throughout the study phases according to the genetic test result status. Status [MT- (mutation) is the presence of a pathogenic variant; WT- (wild type gene) is the absence of a pathogenic variant; VUS- (variant of uncertain significance) is the presence of a variant of unknown significance].

All women encompassed within the study cohort exhibited a discernible concern regarding the potential emergence of novel tumors, a sentiment that persisted from the inception of the study, as depicted in Figure 3. Intriguingly, no statistically significant association was found between GT outcomes and the intensity of their cancer-related worries (*p* > 0.05). Notably, among those who were MT, a consistent trajectory of comparable concern was observed across all four phases of the study. It is, however, crucial to underscore that individuals harboring VUS, in *BRCA1* or *BRCA2*, exhibited an escalating trend of worry spanning the sequential study phases. Conversely, individuals classified as WT experienced a decline in their cancer-related apprehensions. Conversely, pertaining to additional variables such as surgical interventions, educational attainment, familial cancer history, and the number of children, no statistically significant correlations were identified.

### Health belief

Regarding the Health Belief Models (as presented in Supplementary Table S5), notable variations between T0 and T3 were discerned. Within the subset characterized by positive GT outcomes, noticeable increments were evident in the average scores, indicative of heightened perception, pertaining to the potential of cancer development (escalating from 6.7 to 9.9). Moreover, a similar upward trend was observed concerning the perceived advantages associated with preventive strategies for disease detection (rising from 12.9 to 15.8). Conversely, individuals within the VUS category manifested augmented perceptions concerning the gravity of their cancer prognosis (surging from 11.6 to 18.3), as well as the impediments impeding the execution of prevention strategies (increasing from 14.3 to 16.6).

### Coping strategies

The analysis of coping strategies (EMEP) was conducted at both T0 and T3, revealing a recurring trend where individuals with elevated scores demonstrated a propensity for problem-focused coping strategies in conjunction with a preference for religious practices (refer to Supplementary Figure S1). This pattern highlights the participants’ adeptness in navigating challenges while also finding solace in their religious beliefs as a coping mechanism.

To assess how women cope with high cancer risk perception, correlation analysis at T3 (time after completion of CGC and GT) was conducted. Significant correlations were found between the pursuit of religious practices and the perception of risk associated with breast (*p* = 0.014) and ovarian (*p* = 0.015) cancer. This finding emphasizes a notable association wherein heightened perceptions of breast and ovarian cancer risks were accompanied by an increased inclination toward religious engagement as a coping mechanism.

### Anxiety and depression

The participants conspicuously did not exhibit symptoms of anxiety and depression, as evident from Supplementary Figure S2. Notably, it merits highlighting that the mean anxiety score exhibited a consistent decline across all participant groups. Noteworthy is the absence of any statistically significant relationship discerned between this anxiety score and either GT outcomes or the study phases (*p* > 0.05). Regarding depression scores, higher values were observed over time, and statistical significance was established between the scores and the successive study phases (*p* = 0.006). This underscores that passage through the various time points potentially influences the manifestation of depression symptoms.

### Qualitative analysis

An exhaustive evaluation of the genograms and ecomaps was executed during both phases (T0 and T3) through the prism of content analysis. The core theme underpinning the analyses was “Genetic Counseling and GT in Hereditary Breast and Ovarian Cancer,” elucidating five principal categories: (1) “Support and Social Support Network” (which emerged as the pivotal entities women perceived as sources of support); (2) “Attitudes, Feelings, and Emotions” (encompassing women’s reported sentiments, emotions, and concerns); (3) “Cancer Causes” (pertaining to the attributions women held regarding the causative factors of their cancer); (4) “Communication with Relatives” (elaborating on the manner in which women characterized their interactions with family members); and (5) “Relationships with Relatives” (shedding light on how women characterized their familial relationships). Additionally, during T3, an alternate central theme, “Genetic Testing in Hereditary Breast and Ovarian Cancer,” centered on GT, was scrutinized, given the participants’ progression through this experience.

**Figure 4:**
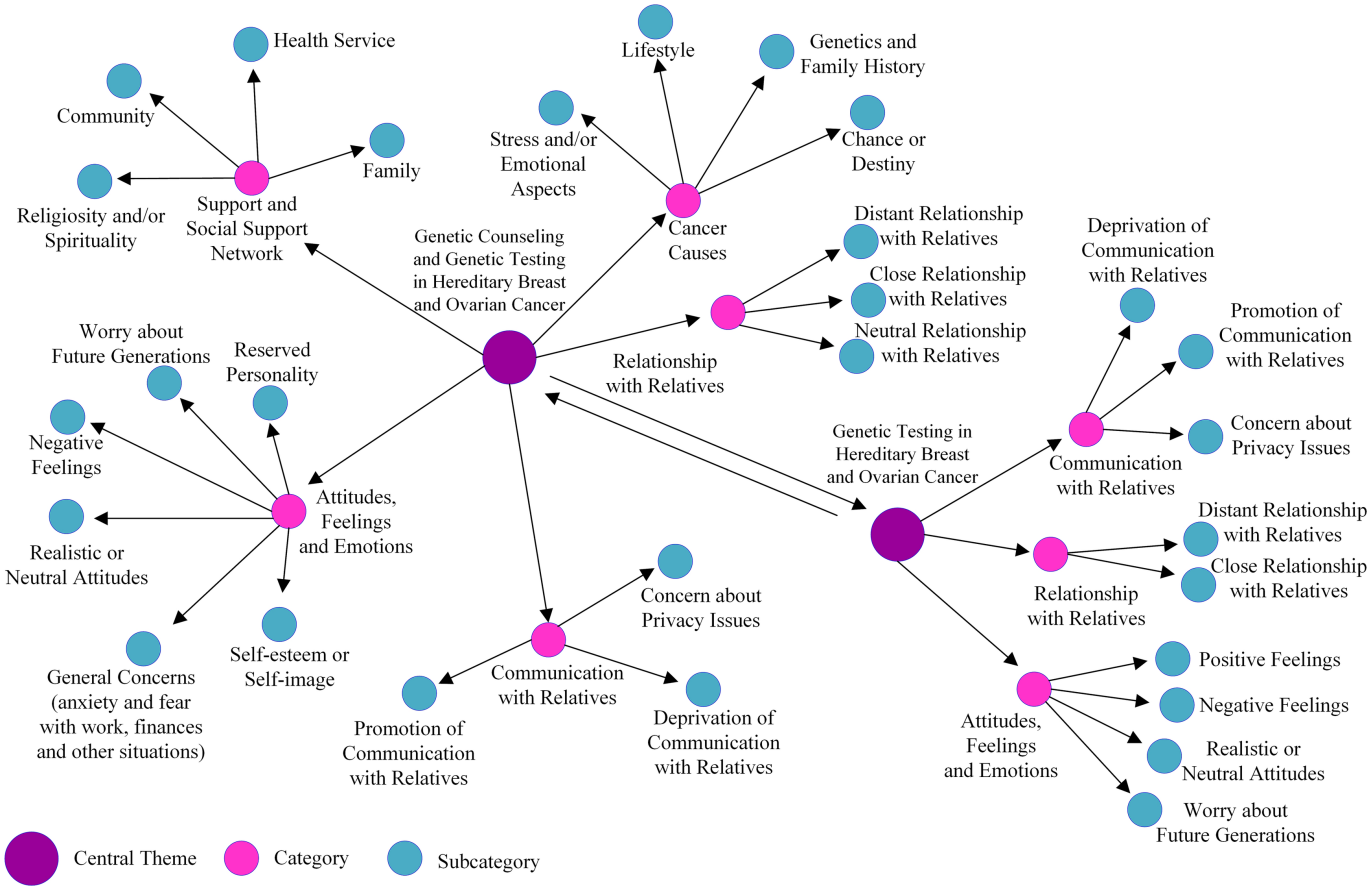
Categorized themes and the respective subcategories according to the central themes “Genetic counseling and genetic testing in hereditary breast and ovarian cancer” for T1 and T4 and “Genetic testing in hereditary breast and ovarian cancer” only for P4.

Elucidated within these categories at T0 and T3 were subcategories, the intricacies of which are depicted in Figure 4. The genograms and ecomaps, aligned with their corresponding identified categories, robustly reinforced the harmony between the identified categories and the outcomes of the semistructured interviews (refer to Supplementary Figure S3).

The category “Support and Social Support Network” prominently featured “Religiosity and/or Spirituality” as the chief subcategory in both phases. Substantial differences in subcategories were evident between T0 and T3 (refer to Supplementary Table S6). The proportion of individuals expressing “Worry about Future Generations” elevated from 18.3 to 35%, whereas “Concern about Privacy Issues” dwindled from 58.3 to 16.6%. A pivotal observation pertains to the substantial surge in reporting “Distant Relationship with Relatives,” ascending from 19 (31.6%) to 29 (48.3%), juxtaposed with a escalation in instances of “Promotion of Communication Among Family Members,” ascending from 9 (15%) to 37 (61.6%).

From a vantage point aligned with the “life course perspective,” scrutinizing age (refer to Supplementary Table S7) unveiled intriguing nuances. Women aged 20 to 29 at T0 demonstrated a paucity of the “Communication with Relatives” category; however, by T3, half of this cohort had embraced the “Promotion of Communication Among Members” subcategory. Another noteworthy revelation from this age bracket was the absence of “Worry about Future Generations” at T0, in contrast to the broader age groups, yet by P4, all individuals within this age range incorporated this subcategory. Women aged 30 years and above, at both time points, converged around “General Concerns” primarily associated with work and financial matters. “Cancer Causes” held greater prominence in all age groups during T0.

Upon segregating participants according to the presence or absence of a family history of cancer (refer to Supplementary Table S8), the group without a family history of cancer exhibited the omission of the “Health Service” subcategory as a facet of social support at both time points. Furthermore, “Communication with Relatives” remained nonexistent at T0 but emerged during T3. Categorizing participants based on GT results (refer to Supplementary Table S10) unveiled intriguing dynamics. Within the MT cohort (16 women), a mere solitary individual identified “Health Service” as a form of social support at T3. Moreover, the “Relationship with Relatives” category revealed an increase in “Distant Relationship with Relatives,” ascending from 25 to 43%, while “Close Relationship with Relatives” declined from 68.7 to 56%. Regarding P4, a pivotal theme of “Genetic Testing in Hereditary Breast and Ovarian Cancer” materialized, wherein the WT (29.2%) and MT (12.5%) cohorts exhibited a preponderance of positive sentiments toward the GT process, an aspect conspicuously absent among carriers of VUS (refer to Supplementary Table S9).

Among all participants, a notable contingent of 12 (20%) individuals—comprising one *BRCA*-WT and 11 MT *BRCA1* or *BRCA2*—accompanied by their family members, attended consultations at the OD. Within this group, 11 (18.3%) brought along at least one first-degree relative, 3 (5%) were accompanied by a second-degree relative, and a sole participant (1.6%) was accompanied by a third-degree relative. Individuals who opted not to bring family members to the OD, comprising 5 participants with pathogenic variants as GT results, revealed genograms and ecomaps indicative of a “Distant Relationship with Relatives,” tethered to specific negative dynamics such as fear, concern, and distrust (refer to Supplementary Table S10).

## Discussion

The process of CGC plays a pivotal role in facilitating the comprehension of genetic conditions for both individuals and their families, primarily through its educational component (Resta et al., 2006). However, the perception of cancer risk constitutes a variable that intertwines with cognitive, social, cultural, and life experiences, thus underscoring the significance of identifying those individuals embarking on these multifaceted processes. This imperative arises from the need to facilitate a meaningful understanding of their circumstances. Notably, concerning GT outcomes and cancer risk perception, our observations revealed that subsequent to the disclosure of GT results (T3), the perception of cancer risk regarding “other cancers” exhibited an escalation among women harboring *BRCA1* and *BRCA2* pathogenic variants, bridging the gap from Phase 2 (post-CGC, but prior to GT result disclosure). This observation may be attributed to this cohort’s antecedent experiences with breast and/or ovarian cancer, fuelling an augmented apprehension toward other malignancies. Though our primary focus was on women with *BRCA* WT or carrying pathogenic alterations, our study inadvertently encompassed 6 *TP53* mutated patients. Notably, *TP53* pathogenic variants confer a heightened susceptibility to diverse cancers spanning both the pediatric and adult spectrum (Achatz and Zambetti, 2016), thus exuding a palpable influence over their risk perception.

Our study unveiled a marked inclination toward religiosity among participants. Importantly, a positive correlation emerged between heightened risk perception of cancer development and an intensified tendency for religiosity. It is paramount to acknowledge that past research has highlighted instances where religious and alternative beliefs may intersect with patients’ medical decision-making processes within the realm of CGC. Thus, the imperative of addressing these beliefs and comprehending individual convictions remains pronounced, despite the attendant challenges (Thompson et al., 2016).

Corroborating with prior research, apprehensions surrounding cancer development increased 6 months post-disclosure of GT results, transcending the outcomes themselves. This pertains to all result categories, irrespective of their specifics. This concordance with preceding studies corroborates the significant escalation in cancer-related concerns in the weeks subsequent to disclosure (Voorwinden and Jaspers, 2016). Notably, patients harboring VUS demonstrated elevated cancer worry scores across the study phases in contrast to those with *BRCA1* and *BRCA2* pathogenic variants or wild-type counterparts. This distinct group grapples with the intricate challenge of navigating an outcome often steeped in ambiguity and uncertainty, eliciting new anxieties (O’Neill et al., 2006). Our study disclosed an additional facet: VUS carriers exhibited elevated scores within the severity and barriers domains. This could be attributed to the absence of personalized cancer prevention and control strategies, possibly precipitating a sense of disillusionment toward conventional screening measures and a perceived absence of protective measures akin to the general populace. This accentuates the imperative of delving into the milieu of uncertainty, underscoring the critical role of the CGC process in its entirety (Medendorp et al., 2021). Importantly, a coherent and thorough educational discourse following the disclosure of a VUS result may avert the emergence of future concerns (Van Dijk et al., 2004).

Regarding anxiety and depression outcomes, a general trend of nominal change pervaded across all study time points. However, at T3, an elevation in depression levels emerged, independent of the GT result. This elevation exhibited a heightened prevalence among MT women in the long term. The interpretation of this finding warrants circumspection, as the assessment of these symptoms relied solely on a single tool that might not comprehensively capture manifestations within this specific cohort of oncogenetic patients (Ringwald et al., 2016). Earlier investigations assessing counselee distress concerning GT underscored the necessity of focusing on emotional dimensions to appraise the requisites for psychological support (Brédart et al., 2022), consequently prompting reflection on the incorporation of tools adept at gauging and assessing pertinent psychological nuances, thus facilitating the fulfillment of specific demands.

It is indispensable to underscore the pivotal role assumed by qualitative analyses, instrumental in engendering a heightened comprehension of the psychosocial characteristics and familial dynamics underpinning the women. These analyses enabled the meticulous assessment of the impacts of CGC and GT within these scenarios. The frequency of category emergence between the initial and final phases of the study (T0 and T3) furnishes pivotal evidence concerning the evolving experiences tethered to CGC and GT. Evidently, the augmentation within the category “Promotion of Communication Among Relatives” from T0 to T3 signifies a heightened degree of intercommunication among family members, regardless of the GT outcome. A prior investigation underscored the multifarious facets influencing communication subsequent to genomic information disclosure, encompassing risk management alternatives, family intimacy levels, and a sense of responsibility (Smit et al., 2021), thus amplifying the centrality of fostering a profound understanding of familial dynamics throughout the CGC process. Strikingly, the study by a North American cohort, encompassing 136 tested patients, underscored that a staggering 96% shared their test results with a family member, accentuating the potential for variable sharing rates contingent on ethnicity (Ricker et al., 2018). This underscores the exigency of investigating communication dynamics within diverse ethnic strata. Evidently, this Brazilian cohort of women resoundingly embraced the role of information sharers, divulging GT results to their family members.

Furthermore, diving into the family peculiarities under the assumptions of the “Life Course Perspective Theory” unveiled a trove of insights. The categorization based on the presence or absence of a familial cancer history unveiled intriguing dynamics. Participants without a familial cancer history exhibited a conspicuous absence of the “Health Service” subcategory within the social support network across both time points. Intriguingly, “Communication with Relatives” surfaced as non-existent at T0, only to manifest during T3. Segmentation of participants based on GT results unraveled nuanced facets. During T3, the crystallization of the central theme “Genetic Testing in Hereditary Breast and Ovarian Cancer” underscored a salient distinction. While WT and MT cohorts were characterized by the prevalence of positive sentiments toward the GT process, this sentiment was conspicuously absent among VUS carriers.

While our study boasts several strengths, foremost among them being the focus on an at-risk population, it is crucial to acknowledge its limitations. The challenge of prospective follow-up lay enshrouded by cases of abandonment and, regrettably, even demise, attenuating the temporal trajectory of these patients and forestalling the consummation of the entire study continuum. Nonetheless, the collective endeavors of the 60 families that traversed all study phases furnished a comparative foundation, thereby illuminating the impacts precipitated by CGC and GT concerning hereditary breast and ovarian cancer.

In conclusion, our study underscores the necessity of delving into the psychosocial milieu and belief systems that shape a particular population. This awareness not only equips healthcare professionals to mitigate potential harm but also highlights the lack of comprehensive insights into the ramifications of CGC and GT within at-risk populations. Knowledge about cancer risk perception, worry about developing cancer, and health belief models is crucial for professionals, enabling them to tailor prevention strategies in an individualized manner.

Besides, our study highlights the crucial need to familiarize oneself with the psychosocial milieu and belief systems of a given population. This awareness not only empowers healthcare professionals to mitigate potential harm but also emphasizes the lack of comprehensive insights into the ramifications of CGC and GT within at-risk populations. Understanding cancer risk perception, concerns about developing cancer, and health belief models can guide professionals in organizing prevention strategies in an individualized manner.

Another crucial aspect involves understanding how women cope with health difficulties, fostering a respectful communication between them and health professionals. This communication strategy aims to encourage comprehension of GT results and effective handling of implications, taking into account religious and other social support systems to minimize potential harms. Equally important is for health professionals to grasp family dynamics and communication within families, as the process of CGC and GT is an educational journey for patients and their families. The methods of transmitting information depend on the understanding acquired during the CGC process, and the family culture significantly influences communication about the events that the family is experiencing (Young et al., 2019; di Pietro et al., 2021).

The identified dearth underscores the urgent necessity for the development of more robust educational and multidisciplinary care strategies. Consequently, meeting the requisites of personalized medicine involves not only technological advancements, including molecular assays and therapeutics, but also personalized care and communication strategies. These measures are essential to ensure that individual and familial care aligns precisely with the unique exigencies of each circumstance.

Considering the limitations of this study, it is crucial to discuss the sample size in relation to the results of germline pathogenic variants (16 women), which represent a significant group in the clinical scenario due to the importance of prospective follow-up and communication with family members. The composition of the sample was influenced by the realities of working within an Oncogenetic Department (OD), which posed challenges in recruiting a larger number of women meeting the inclusion criteria. However, despite this limitation, our study has provided valuable insights into psychosocial aspects and the information obtained from genograms before and after the entire process of Clinical Genetic Counseling (CGC) and Genetic Testing (GT). These findings can assist healthcare professionals in considering personalized approaches in clinical practice, providing support to families undergoing this process.

## Data availability statement

The original contributions presented in the study are included in the article/Supplementary material, further inquiries can be directed to the corresponding author.

## Ethics statement

The studies involving humans were approved by Institutional Ethics Committee from Barretos Cancer Hospital (CAAE: 45128915.6.0000.5437). The studies were conducted in accordance with the local legislation and institutional requirements. The participants provided their written informed consent to participate in this study.

## Author contributions

NC: Conceptualization, Data curation, Formal analysis, Investigation, Methodology, Project administration, Supervision, Writing – original draft, Writing – review & editing. RG: Conceptualization, Methodology, Writing – review & editing. HG: Conceptualization, Writing – review & editing. LG: Conceptualization, Data curation, Investigation, Methodology, Writing – review & editing. PR: Conceptualization, Writing – review & editing. KP: Conceptualization, Writing – review & editing. JG: Conceptualization, Data curation, Methodology, Writing – review & editing. PA-P: Conceptualization, Data curation, Formal analysis, Methodology, Writing – original draft. EP: Conceptualization, Data curation, Formal analysis, Methodology, Project administration, Supervision, Writing – original draft, Writing – review & editing.
