# Supplementary material for: The history of families at-risk for hereditary breast and ovarian cancer: what are the impacts of genetic counseling and testing?
Source: Front Psychol. 2024 Mar 4;15:1306388. doi: 10.3389/fpsyg.2024.1306388 (PMC10946339; doi:10.3389/fpsyg.2024.1306388)
Supplement: Supplementary file 1 [file Data_Sheet_1.docx]

**Supplementary Material**

**Supplementary Table 1:** Psychological Tools

| Variable of evaluation | Tool | Measurement strategy |
| --- | --- | --- |
| Cancer Risk Perception | The Cancer Awareness Needs Survey (CANS) | The CANS questionnaire assesses the perception of breast, ovary, bowel and others (general) and is a cancer risk measure with the options: “Less risk than the general population”, “Equal risk to the general population” and “Higher risk than the general population”. |
| Cancer Worry | The Lerman’s Cancer Worry Scale (CWS) | The CWS has six items and assesses the perceptions of risk and concern about developing cancer, and a score of 0-6 represents minimal worry, 7-13 medium worry and 14-26 maximum worry. |
| Health Belief Model | Champion’s Health Belief Model Scale (CHBM) | This questionnaire evaluates the Health Belief Model using 27 questions divided into four domains: 1) susceptibility (which indicates a greater perception of developing cancer), 2) severity (the severity of cancer to alter physical health), 3) benefits (perceived benefits regarding prevention strategies to detect disease) and 4) barriers (perception that there are negative components of performing the prevention or access difficulties). In each domain, a higher score indicates a greater individual perception of it. |
| Coping Strategies | Ways of Coping Scale (EMEP) | The EMEP is a Brazilian Likert-scale questionnaire composed of 45 items grouped into the following factors: confrontation focused on the problem, focus on emotion, search for religious practices and search for social support. A higher score indicates which factor is more effective for the individual to cope with the problem. These items evaluate thoughts and actions that people use to deal with a specific stressful event |
| Anxiety and Depression | Hospital Anxiety and Depression Scale (HADS) | Analyses of both anxiety and depression scores, with scores from 0 to 8 meaning no symptoms and ≥9 with symptoms |
| Personal and Family History | Genogram and Ecomap | The genogram integrates the biomedical and psychosocial history of the patient and his or her family and allows for the identification of stress factors in the family context. In addition, the ecomap provides an expanded view of the family, designing a support structure and portraying a connection between the family and the world/community. All the genograms and ecomaps were drawn using the software GenoPro 2011. |

**Supplementary Table 2:** Interview guide for genogram and ecomap*.

| Who lives together in the same house with the proband; |
| --- |
| Which relatives have distant relationships; |
| Which family member represents/would represent a caregiver for the proband; |
| Who is the main person in the family for the proband; |
| Which family member do you feel more comfortable talking to; |
| Which family member do you not talk to at all; |
| Is there some person within the family with whom you have a conflicting relationship?; |
| Which family member do you feel most comfortable with?; |
| Who is the provider in your house? (check cases of financial problems and if they cause any imbalance in the family relationship); |
| What is your daily activity? (occupation, check support networks); |
| Do you believe that your family has a good relationship?;; |
| How is talking about problems with your family? (consider changing problems and how family members face them); |
| How is the place where you live? (check home if you have contact with the neighborhood/community as a relief network, for example); |
| Religious aspects (how important is religion; do have traditions and, if so, are they a support network. If you do not have religious beliefs, ask if it is necessary); |
| What person within the family do you consider to have incorrect attitudes?; |
| What is the most urgent concern?; |
| What information family members would like to have and share?; |
| Check places or people who represent support at times with and without a risk of emotional imbalance. |

*Contemplating items during the interview according to Wright LM & Leaheay M, Maximizing time, Minimizing suffering: The 15 minutes (or less) family interview. Journal of Family Nursing (1999). All information was recorded in nursing notes in the participants' medical record.

**Supplementary Table 3** Cronbach alpha of The Lerman’s Cancer Worry Scale from all moments of the study

|  | T0 | T1 | T2 | T3 |
| --- | --- | --- | --- | --- |
| Cronbach α | 0,91 | 0,92 | 0,91 | 0,94 |
| Total de participantes | **60** | **60** | **60** | **60** |

**Supplementary Table 4** Cronbach alpha of Coping Strategies (EMEP) of each domain from all moments of the study

|  | T0 | T3 |
| --- | --- | --- |
|  | Cronbach α | Cronbach α |
| Domain Focused on the problem | 0.74 | 0.64 |
| Domain Focus on emotion | 0.72 | 0.73 |
| Domain Search for religious practice | 0.41 | 0.51 |
| Domain Search for social support | 0.54 | 0.40 |

**Supplementary Table 5**: Scores obtained in the application of the Champion’s Health Belief Model in Phase 1 and 4 by genetic test result status

|  | T0 | | | | T3 | | | |
| --- | --- | --- | --- | --- | --- | --- | --- | --- |
| Escores |  |  |  | **Cronbach α** |  |  |  | **Cronbach α** |
|  | **WT^*^** | **MT^**^** | **VUS^***^** |  | **WT^*^** | **MT^**^** | **VUS^***^** |  |
| Susceptibility (0-20) |  |  |  |  |  |  |  |  |
| -Mean | 8.9 | 6.7 | 8.3 | 0.84 | 8.5 | 9.9 | 8 | 0.93 |
| -Minimum | 5 | 5 | 5 |  | 5 | 5 | 5 |  |
| -Maximum | 16 | 10 | 15 |  | 20 | 16 | 14 |  |
| -SD | 3.4 | 1.7 | 5.7 |  | 4.1 | 4.2 | 5.5 |  |
| Severity (0-28) |  |  |  |  |  |  |  |  |
| -Mean | 15.1 | 16.6 | 11.6 | 0.78 | 16 | 16.8 | 18.3 | 0.78 |
| -Minimum | 7 | 8 | 7 |  | 7 | 9 | 12 |  |
| -Maximum | 26 | 25 | 14 |  | 26 | 25 | 25 |  |
| -SD | 5 | 4.4 | 2.8 |  | 4.7 | 5 | 6.5 |  |
| Benefits (0-20) |  |  |  |  |  |  |  |  |
| -Mean | 14.2 | 12.9 | 15.6 | 0.73 | 16.4 | 15.8 | 15 | 0.73 |
| -Minimum | 9 | 5 | 14 |  | 5 | 7 | 11 |  |
| -Maximum | 20 | 20 | 19 |  | 20 | 20 | 17 |  |
| -SD | 3 | 4.4 | 2.8 |  | 3.6 | 4.1 | 3.4 |  |
| Barriers (0-48) |  |  |  |  |  |  |  |  |
| -Mean | 14.2 | 13.7 | 14.3 | 0.24 | 13.7 | 14.6 | 16.6 | 0.07 |
| -Minimum | 9 | 12 | 14 |  | 12 | 12 | 16 |  |
| -Maximum | 20 | 18 | 15 |  | 19 | 20 | 17 |  |
| -SD | 3 | 2.1 | 0.5 |  | 2 | 1.8 | 0.5 |  |

*Wild type (negative test result) ** Presence of pathogenic variant (positive test result) ***Variant of uncertain significance

Supplementary Table 6: Categories and subcategories identified in the genogram/ecomap at T0 and T3.

| **Categories and Subcategories** | All groups  N/60(%) | | WT^*^  N/41(%) | | MT ^**^  N/16(%) | | VUS^***^  N/3(%) | |
| --- | --- | --- | --- | --- | --- | --- | --- | --- |
|  | T0 | T3 | T0 | T3 | T0 | T3 | T0 | T3 |
| **Support and social support network** |  |  |  |  |  |  |  |  |
| 1-Religiosity and/or spirituality | 54(90) | 53(88.3) | 37(90) | 39(95.1) | 14(87.5) | 11(68.7) | 3 (100) | 3(100) |
| 2-Community | 12(20) | 9(15) | 9(21.9) | 7(17) | 3(18.7) | 1(6.2) | 0 | 0 |
| 3-Family | 12(20) | 2(3.3) | 9(21.9) | 2(4.8) | 2(12.5) | 0 | 1(33.3) | 0 |
| 4-Health service | 4(6.6) | 6(10) | 4(9.7) | 5(12.1) | 0 | 1(6.2) | 0 | 0 |
| **Attitudes, feelings and emotions** |  |  |  |  |  |  |  |  |
| 1- Realistic or neutral attitudes | 8(13.3) | 8(13.3) | 3(7.3) | 5(12.2) | 4(2.5) | 3(18.7) | 1(33.3) | 0 |
| 2- Reserved personality | 3(5) | 2(3.3) | 2(4.8) | 2(4.8) | 1(6.2) | 0 | 0 | 0 |
| 3- General concerns (anxiety and fear over work, finances and other situations) | 32(53.3) | 25(41.6) | 22(53.6) | 18(44) | 10(62.5) | 5(31.2) | 0 | 2(66.6) |
| 4- Worry about future generations | 11(18.3) | 21(35) | 10(24.3) | 14(34.1) | 1(6.2) | 6(37.5) | 0 | 1(33.3) |
| 5- Negative feelings | 15(25) | 2(3.3) | 8(19.5) | 1(2.4) | 6(37.5) | 1(6.2) | 1(33.3) | 0 |
| 6- Self-esteem or self-image | 4(6.6) | 2(3.3) | 2(4.8) | 1(2.4) | 3(18.7) | 1(6.2) | 0 | 0 |
| **Cancer causes** |  |  |  |  |  |  |  |  |
| 1- Stress and/or emotional aspects | 11(18.3) | 0 | 5(12.1) | 0 | 5(31.2) | 0 | 1(33.3) | 0 |
| 2- Chance or destiny | 4(6.6) | 3(5) | 1(2.4) | 2(4.8) | 1(6.2) | 1(6.2) | 2(66.6) | 0 |
| 3- Genetics and family history | 3(5) | 1(1.6) | 2(4.8) | 0 | 1(6.2) | 1(6.2) | 0 | 0 |
| 4- Lifestyle | 6(10) | 0 | 4(9.7) | 0 | 1(6.2) | 0 | 1(33.3) | 0 |
| **Communication with relatives** |  |  |  |  |  |  |  |  |
| 1- Deprivation of communication between members | 5(8.3) | 4(6.6) | 5(12.1) | 3(7.3) | 0 | 0 | 0 | 1(33.3) |
| 2- Promotion of communication among members | 9(15) | 37(61.6) | 7(17) | 24(58.5) | 2(12.5) | 11(68.7) | 0 | 2(66.6) |
| 3- Concern about privacy issues | 5(8.3) | 10(16.6) | 4(9.7) | 6(14.6) | 1(6.2) | 3(18.7) | 0 | 1(33.3) |
| **Relationship with relatives** |  |  |  |  |  |  |  |  |
| 1- Distant relationship with relatives | 19(31.6) | 29(48.3) | 14(34.1) | 21(51.2) | 4(25) | 7(43.7) | 1(33.3) | 1(33.3) |
| 2- Close relationship with relatives | 40(66.6) | 37(61.6) | 27(65.8) | 25(61) | 11(68.7) | 9(56.2) | 2(66.6) | 2(66.6) |
| 3- Neutral relationship with relatives | 1(1.6) | 2(3.3) | 0 | 2(4.8) | 1(6.2) | 0 | 0 | 0 |

*Wild type (negative test result) ** Presence of pathogenic variant (positive test result) ***Variant of uncertain significance.

Supplementary Table 7: Categories and subcategories identified in the genogram/ecomap at T0 and T4 by age group.

| **Categories and Subcategories** | | 20-29  N/4 | | 30-39  N/24 | | 40-49  N/18 | | 50-59  N/8 | | 60-  N/6 | |
| --- | --- | --- | --- | --- | --- | --- | --- | --- | --- | --- | --- |
|  | T0 | | T3 | T0 | T3 | T0 | T3 | T0 | T3 | T0 | T3 |
| **Support and social support network** |  | |  |  |  |  |  |  |  |  |  |
| 1-Religiosity and/or spirituality | 4 | | 4 | 21 | 22 | 16 | 17 | 8 | 6 | 5 | 4 |
| 2-Community | 1 | | 0 | 5 | 3 | 3 | 3 | 2 | 3 | 1 | 0 |
| 3-Family | 1 | | 0 | 4 | 1 | 2 | 1 | 3 | 0 | 2 | 0 |
| 4-Health service | 3 | | 1 | 2 | 3 | 0 | 0 | 0 | 0 | 1 | 2 |
| **Attitudes, feelings and emotions** |  | |  |  |  |  |  |  |  |  |  |
| 1- Realistic or neutral attitudes | 0 | | 1 | 4 | 4 | 3 | 1 | 1 | 1 | 0 | 1 |
| 2- Reserved personality | 0 | | 0 | 0 | 1 | 2 | 1 | 1 | 0 | 0 | 0 |
| 3- General concerns (anxiety and fear over work, finances and other situations) | 1 | | 0 | 18 | 11 | 7 | 9 | 4 | 2 | 2 | 3 |
| 4- Worry about future generations | 0 | | 4 | 5 | 7 | 4 | 5 | 2 | 2 | 1 | 3 |
| 5- Negative feelings | 1 | | 0 | 7 | 2 | 4 | 0 | 4 | 0 | 1 | 0 |
| 6- Self-esteem or self-image | 0 | | 0 | 2 | 2 | 1 | 0 | 1 | 0 | 0 | 0 |
| **Cancer causes** |  | |  |  |  |  |  |  |  |  |  |
| 1- Stress and/or emotional aspects | 0 | | 0 | 5 | 0 | 3 | 0 | 2 | 0 | 1 | 0 |
| 2- Chance or destiny | 0 | | 0 | 1 | 1 | 1 | 2 | 2 | 0 | 0 | 0 |
| 3- Genetics and family history | 1 | | 0 | 1 | 1 | 1 | 0 | 0 | 0 | 0 | 0 |
| 4- Lifestyle | 1 | | 0 | 1 | 0 | 3 | 0 | 1 | 0 | 0 | 0 |
| **Communication with relatives** |  | |  |  |  |  |  |  |  |  |  |
| 1- Deprivation of communication between members | 0 | | 0 | 3 | 2 | 1 | 1 | 0 | 1 | 1 | 0 |
| 2- Promotion of communication among members | 0 | | 2 | 4 | 14 | 2 | 4 | 1 | 4 | 2 | 5 |
| 3- Concern about privacy issues | 0 | | 0 | 3 | 4 | 1 | 2 | 1 | 2 | 0 | 2 |
| **Relationship with relatives** |  | |  |  |  |  |  |  |  |  |  |
| 1- Distant relationship with relatives | 1 | | 2 | 8 | 12 | 4 | 1 | 4 | 1 | 2 | 3 |
| 2- Close relationship with relatives | 3 | | 2 | 15 | 13 | 14 | 6 | 3 | 6 | 1 | 4 |
| 3- Neutral relationship with relatives | 0 | | 0 | 1 | 1 | 0 | 0 | 1 | 0 | 3 | 1 |

Supplementary Table 8: Categories and subcategories identified in the genogram/ecomap at T0 and T3 considering the presence or absence of family history of cancer.

| **Categories and Subcategories** | Without Family History of cancer  N/4 (%) | | With Family History of  cancer  N/56 (%) | |
| --- | --- | --- | --- | --- |
|  | T0 | T3 | T0 | T3 |
| **Support and social support network** |  |  |  |  |
| 1-Religiosity and/or spirituality | 4(100) | 3(75) | 50(89.2) | 50(89.2) |
| 2-Community | 0 | 1(25) | 12(21.4) | 8(14.2) |
| 3-Family | 1(25) | 0 | 11(19.6) | 2(3.5) |
| 4-Health service | 0 | 0 | 4(7.1) | 6(10.7) |
| **Attitudes, feelings and emotions** |  |  |  |  |
| 1- Realistic or neutral attitudes | 0 | 1(25) | 8(14.2) | 7(12.5) |
| 2- Reserved personality | 0 | 0 | 2(3.5) | 2(3.5) |
| 3- General concerns (anxiety and fear over work, finances and other situations) | 2(50) | 1(25) | 30(53.5) | 24(42.8) |
| 4- Worry about future generations | 0 | 1(25) | 11(19.6) | 19(33.9) |
| 5- Negative feelings | 0 | 0 | 14(25) | 2(3.5) |
| 6- Self-esteem or self-image | 0 | 0 | 4(25) | 2(3.5) |
| **Cancer causes** |  |  |  |  |
| 1- Stress and/or emotional aspects | 0 | 0 | 11(19.5) | 0 |
| 2- Chance or destiny | 1(25) | 0 | 3(5.3) | 3(5.3) |
| 3- Genetics and family history | 0 | 0 | 3(3.3) | 1(1.7) |
| 4- Lifestyle | 0 | 0 | 6(10.7) | 0 |
| **Communication with relatives** |  |  |  |  |
| 1- Deprivation of communication between members | 0 | 0 | 5(8.9) | 4(25) |
| 2- Promotion of communication among members | 0 | 4(100) | 9(16) | 33(59) |
| 3- Concern about privacy issues | 0 | 0 | 5(8.9) | 10(17.8) |
| **Relationship with relatives** |  |  |  |  |
| 1- Distant relationship with relatives | 1(25) | 2(50) | 18(32.1) | 27(48.2) |
| 2- Close relationship with relatives | 3(75) | 2(50) | 37(66) | 34(60.7) |
| 3- Neutral relationship with relatives | 0 | 0 | 1(1.7) | 2(3.5) |

Supplementary Table 9: Categories and subcategories identified in the genogram/ecomap at T3 considering the theme “Genetic testing in breast and ovarian cancer” according the genetic test result.

| **Categories e subcategories** | WT^*^  N/41(%) | MT ^**^  N/16(%) | VUS^***^  N/3(%) |
| --- | --- | --- | --- |
|  |  |  |  |
| **Attitudes, feelings and emotions** |  |  |  |
| 1- Realistic or neutral attitudes | 4 (9.7) | 0 | 0 |
| 2- Negative feelings | 2 (4.8) | 2 (12.5) | 2 (66.6) |
| 3-Positive feelings | 12 (29.2) | 2 (12.5) | 0 |
| 4- Worry about future generations | 0 | 7 (43.7) | 0 |
| **Communication with relatives** |  |  |  |
| 1- Deprivation of communication between members | 1 (2,4) | 1 (6.2) | 0 |
| 2- Promotion of communication among members | 24 (58.5) | 9 (56.2) | 1 (33.3) |
| 3- Concern about privacy issues | 0 | 1 (62) | 0 |
| **Relationship with relatives** |  |  |  |
| 1- Distant relationship with relatives | 1 (2.4) | 4 (25) | 0 |
| 2- Close relationship with relatives | 3 (7.3) | 2 (12.5) | 0 |

*Wild type (negative test result) ** Presence of pathogenic variant (positive test result) ***Variant of uncertain significance.

**Supplementary Table 10:** Characterization of the changes of the families with pathogenic variants (MT) in which the relatives did not come to the consultations in the oncogenetics department until T3.

|  | **Gene with PV*** | **Appearance of Relative at P4** | **Comments** |
| --- | --- | --- | --- |
| **Family** | | | |
| **F02** | *BRCA1* | Conflict | Son does not want to perform the genetic test, but the mother (proband) insists. |
| **F29** | *BRCA1* | Concerns | Concerns regarding the relationship with oncogenetics decision making and with future generations |
| **F85** | *BRCA1* | Impoverished Relationship | After the death of the proband’s mother, there was a separation of the relatives. |
| **F90** | *BRCA1* | Fear | Family fears the genetic counseling appointment because they do not know what will happen at the oncogenetics department. |
| **F34** | *TP53* | Distrust and Conflict | The proband no longer trusts the hospital since the professionals did not find his or her cancer recurrence early. The proband's nieces wants to take the test, but her parents do not think it is a good idea. |

*Pathogenic variant.
