# Supplementary figures and images for: The history of families at-risk for hereditary breast and ovarian cancer: what are the impacts of genetic counseling and testing?
Source: Front Psychol. 2024 Mar 4;15:1306388. doi: 10.3389/fpsyg.2024.1306388 (PMC10946339; doi:10.3389/fpsyg.2024.1306388)

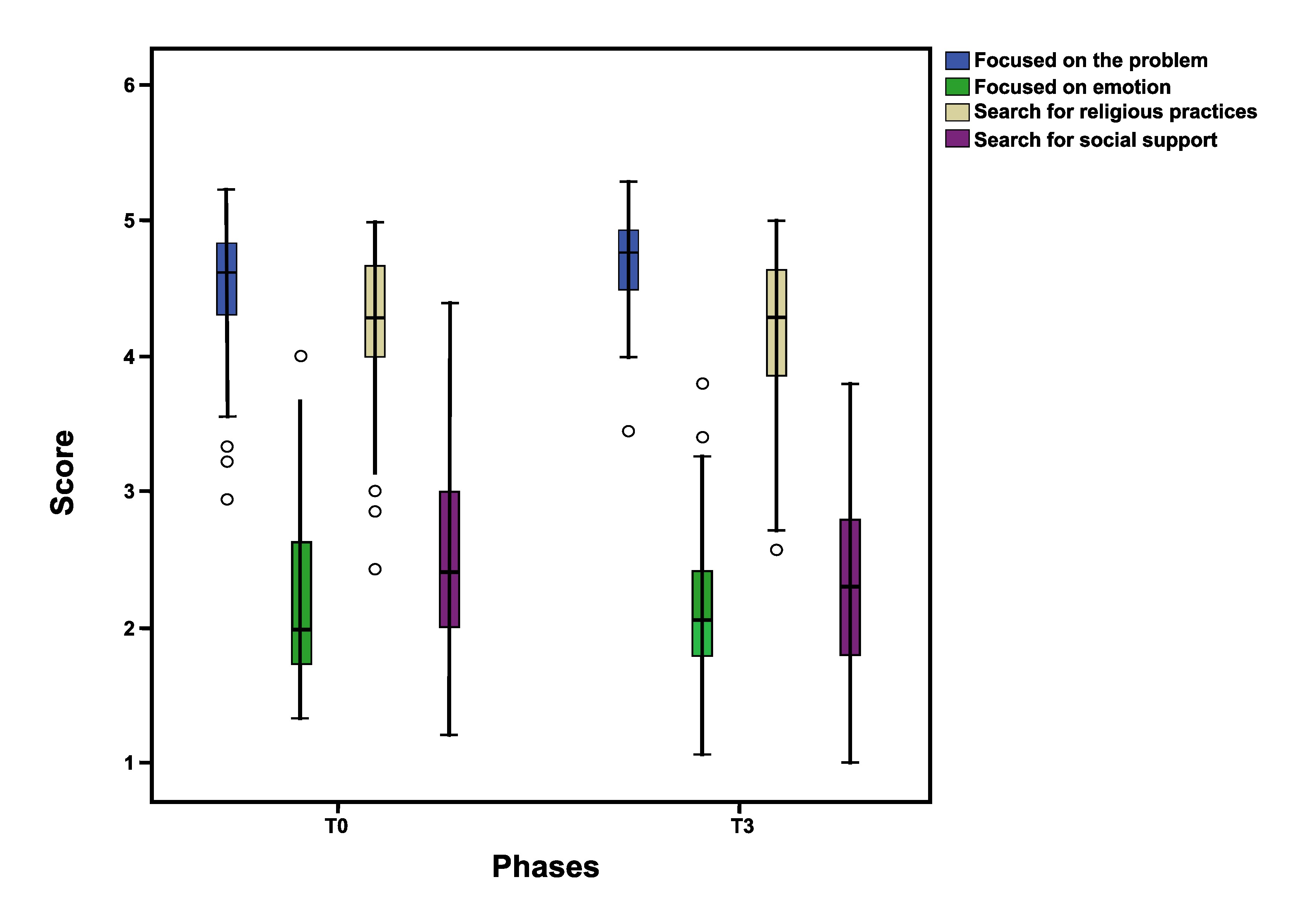

Supplement: Supplementary file 2 [file Image_1.JPEG]

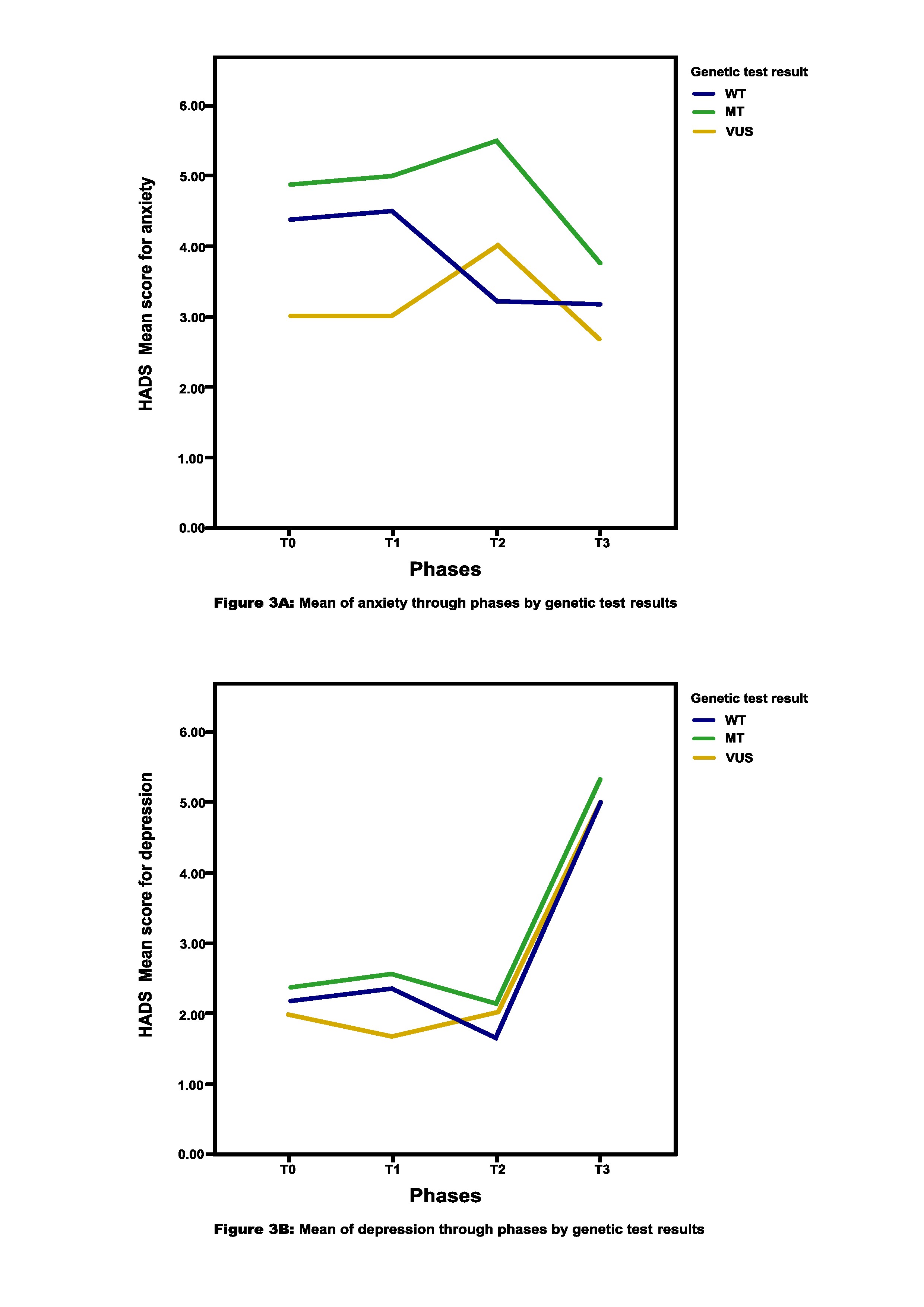

Supplement: Supplementary file 3 [file Image_2.JPEG]

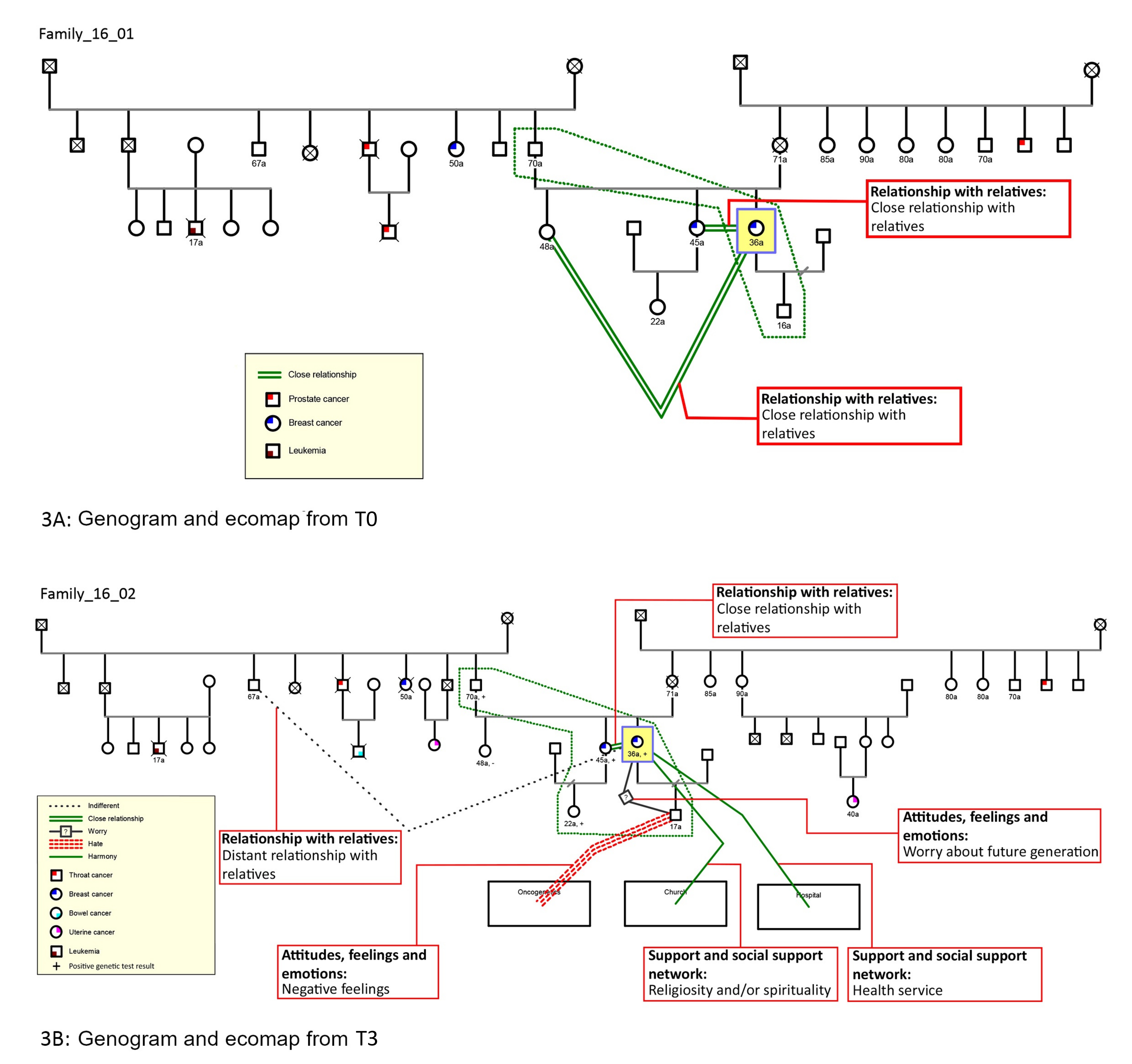

Supplement: Supplementary file 4 [file Image_3.JPEG]
